# Prevalence and factors associated with sarcopenia among urban and rural Indian adults in middle age: A cross-sectional study from Western India

**DOI:** 10.1371/journal.pgph.0003553

**Published:** 2024-10-01

**Authors:** Gauri Bhat, Alex Ireland, Nikhil Shah, Ketan Gondhalekar, Rubina Mandlik, Neha Kajale, Tarun Katapally, Jasmin Bhawra, Rahul Damle, Anuradha Khadilkar

**Affiliations:** 1 Hirabai Cowasji Jehangir Medical Research Institute, Jehangir Hospital, Pune, Maharashtra, India; 2 Department of Health Sciences, Savitribai Phule Pune University, Pune, Maharashtra, India; 3 Department of Life Sciences, Research Centre for Musculoskeletal Science & Sports Medicine, Manchester Metropolitan University, Manchester, United Kingdom; 4 Division of Pediatric Endocrinology, MRR Children’s Hospital, Thane, Maharashtra, India; 5 DEPtH Lab, School of Health Studies, Faculty of Health Sciences, Western University, London, Ontario, Canada; 6 Department of Epidemiology and Biostatistics, Schulich School of Medicine and Dentistry, Western University, London, Ontario, Canada; 7 Children’s Health Research Institute, Lawson Health Research Institute, London, Ontario, Canada; 8 School of Occupational and Public Health, Toronto Metropolitan University, Toronto, Canada; 9 Department of Orthopaedics, Jehangir Hospital, Pune, Maharashtra, India; Universiti Malaya, MALAYSIA

## Abstract

Sarcopenia is the age-related loss of muscle mass and function. India has 8.6% of the global elderly (>60 years) population, and this is expected to increase to 20% by 2050. Around 70% of Indians live in rural areas where lifestyle factors like diet and physical activity differ from urban areas. Understanding age, sex and location-specific sarcopenia prevalence in India is crucial. Thus, our aim was to assess the prevalence and determinants of sarcopenia in urban and rural community-dwelling men and women aged 40 years and older, representing the next generation of older Indian adults. This cross-sectional study included 745 adults (400 women) from urban and rural areas near Pune, Western India. Assessments included socio-demography, diet by-24-hr recall, physical activity, anthropometry (height, weight), muscle mass measurement by dual-energy X-ray absorptiometry, muscle strength (hand grip) & muscle function by Short Physical Performance Battery (SPPB). Sarcopenia was defined by Asian Working Group on Sarcopenia-2019 guidelines Mean age of participants was 53±7.6yrs. Overall prevalence of sarcopenia was 10% and of severe sarcopenia was 4.2%. Sarcopenia prevalence was higher in rural (14.8%) than urban (6.8%) participants and in men (12.5%) than women (8%, all *p*<0.05). Muscle mass, grip strength and SPPB score were all higher in urban than rural participants (*p*<0.05). Older age, rural residence, inadequate protein intake, and lower socio-economic status were independently associated with sarcopenia. In this middle-aged group, sarcopenia prevalence was similar to that observed in older Western populations, over 100% higher among rural than urban participants, and higher amongst men than women. Age, location, protein intake and socioeconomic status were factors associated with sarcopenia. Given this rapidly increasing population of older adults in India there is an urgent need to plan strategies for early sarcopenia diagnosis and management, especially in rural populations.

## Introduction

During the human ageing process, there is a progressive decline in skeletal muscle mass and function, a condition known as sarcopenia [1–3]. There is approximately a 30% loss of muscle mass between the ages of 20–80 years [3]. Sarcopenia leads to a decline in functional capacity, increased risk of falls and fractures, physical disability, increased frailty syndrome, poor quality of life and even premature death [3, 4]. Due to these adverse outcomes, sarcopenia is a major public health problem, recognition of which is indicated by the assignment of an International Classification of Diseases (ICD-10-Rev:10,2016) code for sarcopenia [5].

The aetiology of sarcopenia is multifactorial including factors like age, socio-demography, lifestyle, genetic, hormonal, and certain health conditions [2, 6]. Muscle mass decreases at an annual rate of approximately 1–2% and muscle strength declines at 1.5%, with losses increasing after age 60 with a higher rate reported in sedentary individuals and in men than women [7]. This indicates that men and women show different trajectories of decline in muscle parameters with ageing [7]. Women show an accelerated decline of muscle mass and strength around and post-menopause in part due to low serum estrogen concentrations [8]. Studies have shown that there are age-related differences in prevalence of sarcopenia and also that there are sex-specific differences in muscle loss rates among men and women indicating age and sex-specific risk factors exist among sarcopenic individuals [9]. Hence, understanding of sex- and age-specific sarcopenia risk is crucial for interventions and healthcare planning.

Asia is the fastest ageing region in the world. The proportion of Asian citizens over 60 years is expected to increase to 10% by 2025 and 19% by 2050 and in the coming years, sarcopenia will have a greater impact on Asian populations than other regions [10]. India has the second highest elderly (>60 years) population in the world with 8.6% of the global share in 2011, this is expected to increase to 20% by the year 2050 [11]. Around 71% of the population in India live in rural areas, where important risk factors for sarcopenia including poor diet and physical inactivity differ from urban areas [12].

Due to rapid demographic and epidemiological transition and urbanization, complex changes are occurring in the lifestyles in most of the developing world, including India [13]. Although Asian populations have a more physically active lifestyle than in western populations, they have a relatively smaller body size, higher adiposity, and lower muscle mass [14]. Further to this, there are substantial differences in socio-economic environment, lifestyle factors and health-care resources between urban-rural areas [15]. These differences influence the health outcomes of elderly, resulting in poorer outcomes in rural adults than urban ones [16]. Along with nutritional differences, physical activity levels in India have experienced a secular decline due to increasing urbanization, and economic growth [13, 14].

Given the cumulative effect of prolonged exposure to poor diet and physical inactivity, identification, and subsequent intervention with individuals at risk of sarcopenia in midlife has potential for greater improvements in later life function and health. Despite this high burden, there is limited information on sarcopenia and its determinants in middle-aged and older Indian adults, and no comparisons have been reported between urban and rural individuals. Also, due to a lack of normative data, there are challenges in diagnosis and there is likely to be an underestimation of the prevalence of sarcopenia [17–19]. There is thus a need for comprehensive community-based studies to assess the burden of the condition.

In 2019, a revised consensus by the European Working Group on Sarcopenia in Older People (EWGSOP2) was published with primary focus on low function. Participants with low muscle mass (low quantity/quality), low muscle strength and function are considered to have severe sarcopenia [20]. Similar guidelines are followed by the Asian Working Group for Sarcopenia (AWGS) which supports early identification of people at risk to enable timely intervention and promoting sarcopenia research in Asian countries by providing diagnostic cut-off values based on Asian studies [21].

Taken together, our aim was to assess the prevalence of sarcopenia in urban and rural community dwelling Indian middle-aged adults above 40 years of age. Our secondary objective was to determine modifiable factors associated with sarcopenia in this population, to identify potential opportunities for intervention.

## Methods

### Study design and sample size

This was a prospective, cross-sectional study conducted among urban and rural men and women above 40 years of age.

### Ethical approval

This study was approved by Institutional Ethics Committee, Jehangir Clinical Development centre (JCDC)and written informed consent was obtained from all participants. (Approval date: 2/03/2020)

Based on 17% prevalence of sarcopenia reported from a global study by Tyrovolas et al [22], using the definition of sarcopenia as low skeletal muscle mass and either slow gait speed or low hand grip strength, and considering 4–5% variation among the population, a total sample size was calculated with the formula (n = Z^2^ * P(1-P)/e^2^) as 350 urban and 350 rural with 175 men and 175 women from each location.

### Inclusion and exclusion criteria

**Fig 1:**
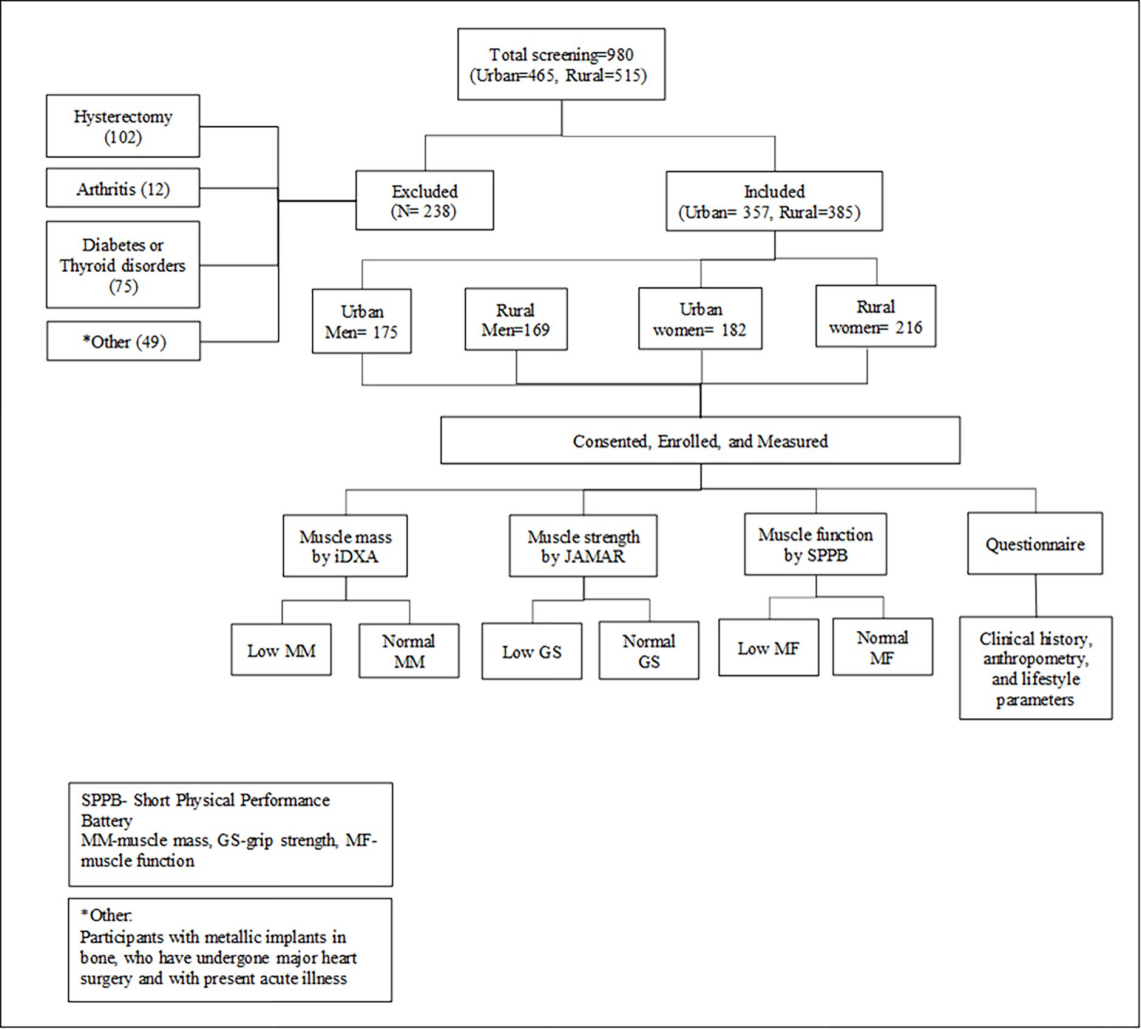
Flow chart for participant enrolment from urban and rural areas.

Apparently healthy men and women above age 40 years and who consented to participate were included. Rural participants were recruited between 20-06-2020 and 25-02-2022, whilst urban participants were recruited between 27-05-21 and 21-03-23. Participants with present acute illness, co-morbidities like diabetes, thyroid disorders, arthritis, and chronic conditions (liver/kidney failure, cardio-vascular disease, etc.) & with major surgeries were excluded. Participants with any metal implant in their bones and women who were pregnant/lactating, who had undergone hysterectomy were excluded from the study (**Fig 1**).

Data on age, sex, location, socio-economic status [23], medical and reproductive history (in women) were obtained using a structured and pre-tested questionnaire (intra class correlation coefficients 0.81–0.99). Data on history of past and current illness, medications, and reproductive history in women (age at menarche, marital status, parity, age at menopause) was obtained using a structured questionnaire. Height was measured using a stadiometer (Seca-Portable stadiometer, Hamburg, Germany), weight was measured using a Bioelectrical Impedance Analyzer (BIA) weight scale, and body mass index (BMI) was calculated.

### Lifestyle factors

The Global Physical Activity Questionnaire (GPAQ) [24] was adapted and validated for an Indian urban and rural population to capture physical activity data. Activities were classified as job or occupation related, household and recreational. Information about time spent in minutes for daily activities like work, household activities, exercise/recreational, sleep, sitting, travelling was obtained and activities were classified as heavy, moderate, and light activities. These activities were classified using MET scores as described in the Compendium of Physical Activity (Version-2.) [25]. The participant was considered as physically active if they performed moderate physical activity for more than 150 min/week or vigorous physical activity for more than 75 min/week as per WHO guidelines [26].

Sunlight exposure was assessed by using a validated questionnaire and was considered adequate if the participant was exposed to sunlight equivalent to >2 hours of casual sunlight exposure daily with 15% skin surface exposed between 11am- 3 pm [27].

Dietary intake was assessed by 24-hr recall by a trained investigator using standard cups and spoons. Nutrient intake was computed using C-diet software ver. 3.2 (cooked and raw food database software) [28]. As per ICMR-NIN guidelines for Nutrient Requirement for Indians, estimated Average Requirements (EAR) were referred to for intake of protein (0.66gm/kg/day i.e. 43g protein/day for men weighing 65 kg and 36g protein/day for women weighing 55kg) and participants in which protein consumption was less than EAR were referred as having inadequate protein intake [29].

### Measurement of muscle parameters

AWGS-2019 recommends the use of dual-energy X-ray absorptiometry (DXA) (height adjusted) for measurement of muscle mass in sarcopenia diagnosis [20]. Hence as per the protocol, in the present study muscle mass was measured using DXA (Lunar-iDXA GE Healthcare, Encore V-16) to assess total and appendicular skeletal muscle mass (ASM) and body composition and Appendicular skeletal muscle index (ASMI-kg/m^2^) was calculated as ASM/ (height in m^2^) [30, 31].

Participants were positioned for a whole-body scan according to the manufacturer’s protocol. In order to minimize interobserver variation, all scans and analyses were carried out by the same technician and the coefficient of variation (CV) for total body lean mass was <1% [30]. The machine was calibrated by performing quality assurance (QA) checks daily.

AWGS 2019 recommends the use of hand grip strength to measure muscle strength using either a spring type (Smedley) or hydraulic type (JAMAR) hand dynamometer using standard protocols [20]. In this study, grip strength was measured using a hydraulic type hand dynamometer (JAMAR-Plus Hand dynamometer, Warrenville, IL, USA).

The American Society of Hand Therapists and the Southampton protocol recommends sitting with 90-degree elbow flexion as the standard position for measuring handgrip strength using the Jamar dynamometer [20]. A similar protocol was used in our study; the participant was seated in the upright position and the dynamometer handle was adjusted to accommodate participants’ hand size and hand grip was measured. The grip strength was measured for both hands. Three readings with a rest of 30-seconds between each reading were recorded and the average was considered for the assessment.

### 3. Measurement of muscle function

Published studies have used a wide range of physical performance tests which include usual gait speed test, 6m-walk test, short physical performance battery (SPPB), five-time chair-stand test etc. AWGS-2019 recommends defining low physical performance based on either SPPB, 6-m walk, or five-time chair stand test cutoffs [20].

In this study, muscle function was assessed using the Short Physical Performance Battery (SPPB) explained by *Guralnik et al* [32]. This includes three simple tests: the standing balance test, gait speed test and five-times chair stand test: All the tests were scored from 0 to 4 using standard thresholds and summed for a total score ranging from 0 to 12; higher scores indicate better muscle function.

### Diagnosis of sarcopenia

Sarcopenia was defined as age-related loss of skeletal muscle mass and loss of muscle strength and/or reduced physical performance without any co-morbidity as per the AWGS-2019 revised guidelines. Specifically, AWGS 2019 introduces “possible sarcopenia,” defined by low muscle strength with/without reduced physical performance. To define low muscle mass, AWGS-2019 recommends use of cutoffs for low muscle mass as less than 7.0kg/m^2^ for men and 5.4kg/m^2^ for women by DXA [20].

AWGS-2019 does not propose dynamometer-specific cut-offs and recommends low muscle strength diagnostic-cut-offs of handgrip less than 28.0 kg for men and less than 18.0kg for women [20].

SPPB score ≤ 9 as the cut-off or low physical performance was used in which performance of balance test for 10 seconds, 5-timechair stand time cut-off of 12 seconds and gait speed cut-off as 0.8m/sec were used [20].

### Statistical analysis

Statistical analyses were carried out using the IBM-SPSS Statistics for Windows-(version 21.0, IBM Corp, Armonk, NY, USA); study parameters were tested for normality. Continuous variables of demographic, anthropometric, muscle and lifestyle parameters between urban-rural participants were compared using T-test and categorical variables were compared by column proportions. Binary logistic regression models were used to determine factors associated with sarcopenia.

## Results

**Table 1** illustrates the comparison of descriptive characteristics between urban and rural men and women.

**Table 1:** Descriptive characteristics of urban and rural participants.

Characteristics	Urban Men (N = 176)	Rural Men (N = 169)	Urban Women (N = 184)	Rural Women (N = 216)	Total Men (N = 345)	Total Women (N = 400)
**Demographic Characteristics**						
Age (yrs)	53.5±7.7	53.8±7.7	52.7 ±7.7	53.2±7.5	52.3±7.8	52.9±7.8
**Socio-Economic Status**						
High SEC n (%)	145 (82.4) *^a^	71 (42.0)	173 (94.0) *^b^	91 (42.1)	216 (62.6)	264 (66.0)
Low/Middle SEC n(%)	31 (17.6)	98 (58.0)	11 (6.0)	125 (57.9)	129 (37.4)	136 (34.0)
**Anthropometric Parameters**						
Height (cm)	168.1±6.1*^a^	165.1±5.6	153.3±5.5	151.7±5.9	166.6±6.1*^c^	152.5±5.8
Weight (Kg)	72.5±11.1*^a^	66.1±11.9	64.9±10.5*^b^	56.9±11.4	69.3±11.9*^c^	60.6±11.7
BMI (Kg/m^2^)	25.6±3.6*^a^	24.1±3.9	27.7±4.2*^b^	24.7±4.7	24.9±3.7*^c^	26.0±4.7
MUAC (cm)	29.4±3.1*^a^	27.4±3.1	29.7±3.5*^b^	26.9±3.5	28.4±3.2	28.2±3.7
WC (cm)	90.9±9.7	88.3±10.2	86.9±9.5*^b^	78.2±10.7	89.6±10.0*^c^	82.2±11.0
CC (cm)	35.6±3.2*^a^	32.5±3.2	35.3±3.4*^b^	31.0±3.4	34.1±3.6*^c^	32.9±4.0
**Muscle parameters**						
ASMI (Kg/m^2^)	7.8±0.9*^a^	7.4±0.9	6.7±0.8*^b^	6.1±0.8	7.6±0.9*^c^	6.4±0.9
Grip strength (Kg)	31.1±6.2*^a^	25.6±6.5	18.9±4.8*^b^	15.5±3.8	28.4±6.9*^c^	17.1±4.6
SPPB score	11.1±1.5	10.8±1.4	11.08±1.2*^b^	10.03±2.0	10.9±1.4*^c^	10.5±1.8
**Lifestyle parameters**						
**Dietary intake**						
Energy (Kcal/day)	1759±380*^a^	1473±419	1514±342*^b^	1131±356	1620±424*^c^	1307±398
Protein (gm/day)	42±15*^a^	36±14	36±13*^b^	26±10	39±15*^c^	30±13
Carbohydrates(gm/day)	290±59*^a^	242±68	248±56*^b^	190±55	266±68*^c^	217±63
Fat (gm/day)	47±18*^a^	39±17	40±15*^b^	28±14	43±18*^c^	34±16
Protein adequacy n (%)	69 (39.2) *^a^	36 (21.3)	88 (47.8) *^b^	25 (11.6)	105 (30.4)	113 (28.3)
**Physical activity**						
Heavy (min/week)	0(0) *^a^	240(2520)	0(0) *^b^	0(2520)	0 (390)	0(73)
Moderate (min/week)	2625(2775) * ^a^	360(2073)	807(2175) *^b^	777(863)	1225(2703)*^c^	790(1256)
Light (min/week)	415(661)	210(788)	1452(903) *^b^	1120 (901)	325(765) *^c^	1260(882)
**Sunlight exposure**						
Sunlight exposure(min)	20(35) * ^a^	112(158)	15(23) *^b^	62(158)	40(17,121)	30(10,90)
Adequate sunlight n(%)	13 (7.4) *^a^	81 (47.9)	3 (1.6) *^b^	77 (35.6)	94(27.2) *^c^	80 (20.0)
**Tobacco use**						
Currently smoking/tobacco use	31(17.6) *^a^	90 (53.3)	12 (6.5) *^b^	83 (38.4)	121(35.1) *^c^	95 (23.8)

All values represented as Mean±S.D or n (%), Physical activity and sunlight exposure are presented as median (IQR)

*All p values <0.*05*

a-significantly different than rural men

b-significantly different than rural women

c-significantly different than women.

SEC-socio-economic class, BMI-Body mass index, MUAC-Mid-upper arm circumference, WC- waist circumference, TSFT- Triceps skinfold thickness, CC-Calf circumference, ASMI- Appendicular skeletal muscle index, SPPB-Short Physical Performance Battery. Protein adequacy presented as per EAR guidelines. Sunlight exposure adequacy as described by Patwardhan et al.

### Socio-demographic characteristics

A total of 745 participants, 360 urban and 385 from rural areas participated (345 men and 400 women). There was no significant difference in age between urban and rural participants (*p*>0.05). There was a significant difference in the socio-economic status between urban and rural participants (*p*<0.05). Most of the urban participants were from the higher socio-economic class and rural participants were from lower or middle class [23].

### Anthropometric parameters

Urban men and women were taller and heavier than their rural counterparts (*p*<0.05). Indicators of adiposity (BMI, WC) and muscle mass (MUAC & CC) were higher in the urban population (*p*<0.05).

### Muscle parameters

Mean values of muscle parameters such as muscle mass (expressed as appendicular skeletal muscle index), grip strength and muscle function score (given by SPPB) were significantly higher among urban participants than their rural counterparts (p<0.05, **Table 1).**

### Lifestyle parameters

Nutrient intake differed between the urban and rural participants (*p*<0.05). Total energy, protein, carbohydrate, and fat intake was significantly higher among urban participants than their rural counterparts. As per Indian guidelines (ICMI-NIN-2021) [29] for Estimated Average Requirement (EAR), only 29% of total participants had an adequate protein intake among which, 16% from rural (men = 21%, women = 12%) and 44% from urban (men = 39%, women = 48%) reported adequate protein intake (*p*<0.05). Protein adequacy did not differ significantly among men and women.

Rural men and women were involved in more moderate to heavy occupational activities (farming, animal care) than their urban counterparts, while urban men and women took part in more moderate to light physical activity (*p <0*.*05)*).

Sunlight exposure was higher in rural than urban residents (41% vs 7%) and it was reported higher in rural men (48%) than urban men (7%) and in rural women (36%) than urban women (2%) (all p<0.05).

Rural men reported higher tobacco consumption/smoking than urban men (53% vs 17%) and rural women reported higher tobacco use than urban women (38% vs 7%). (*All p<0*.*05)*

**Fig 2:**
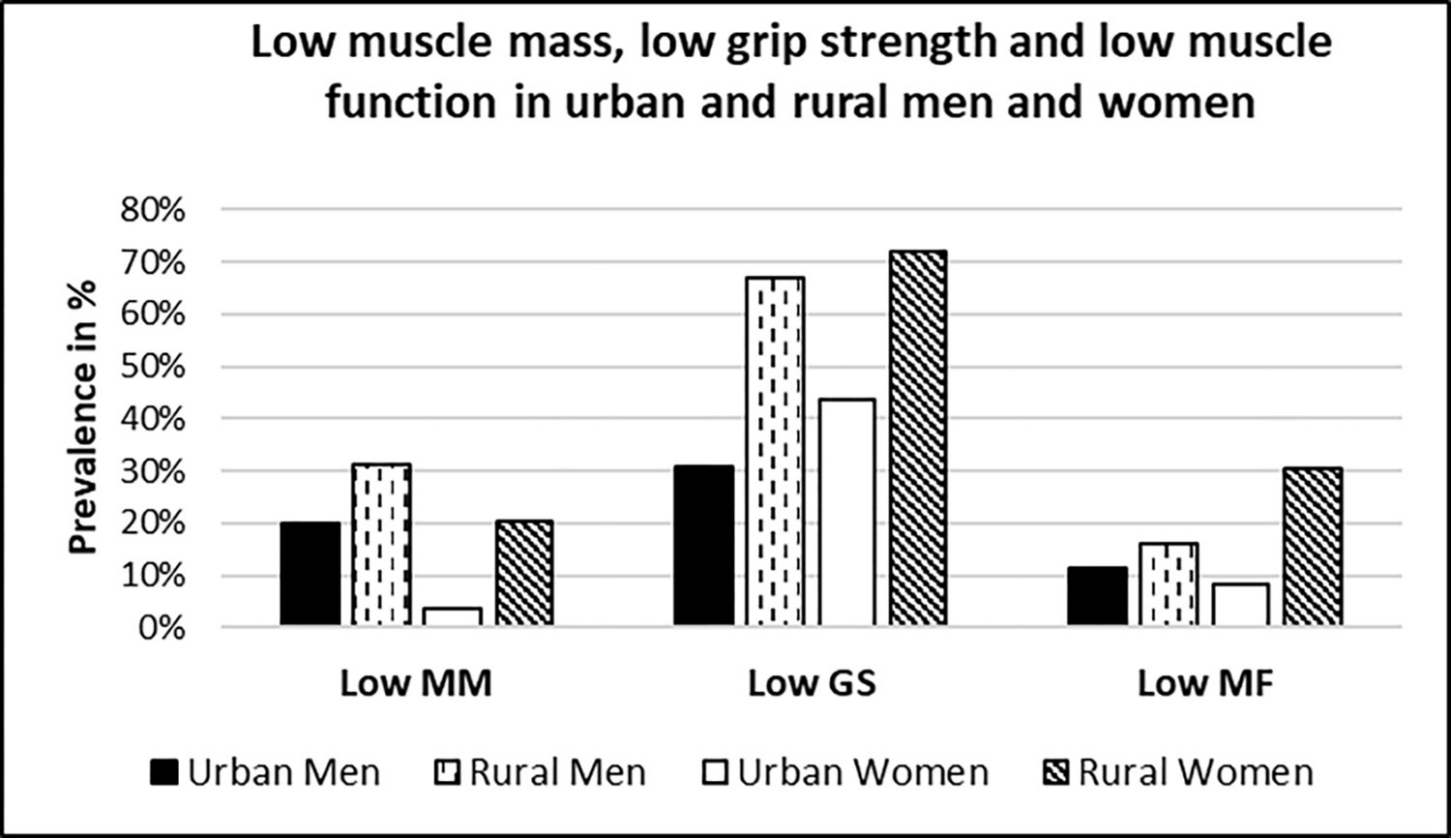
Prevalence of low muscle mass, low grip strength and low muscle function among urban and rural population. MM-Muscle mass, GS- Grip Strength, MF- Muscle Function. Urban men (n = 175), Rural Men (n = 169) Urban Women (n = 182) Rural women (n = 216).

**Fig 2.** illustrates the prevalence of low muscle mass, low muscle strength and low muscle function among the urban-rural participants. Rural participants as compared to urban, showed a higher proportion of low muscle mass (20% vs 31%), low grip strength (67% vs 72%) and low muscle function (16% vs 31%).

**Fig 3:**
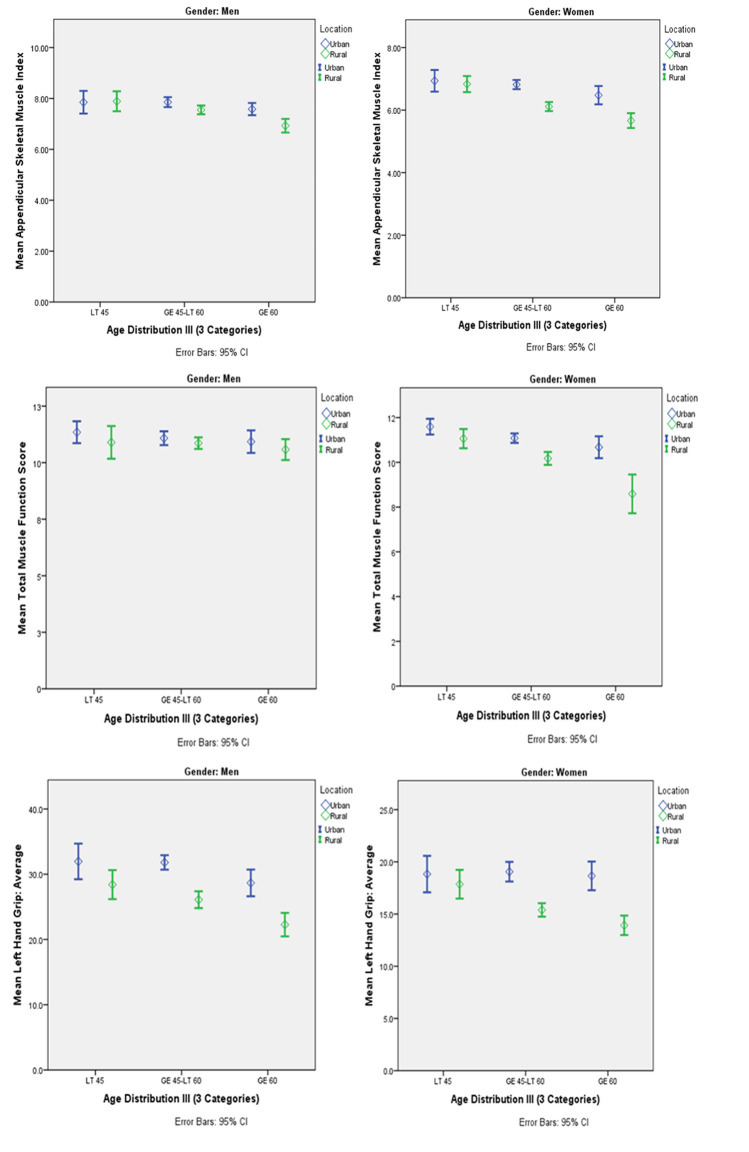
Muscle parameters among men and women split by location (urban/rural) and age group. (Data are presented as mean and 95%CI). Urban men (n = 175), Rural Men (n = 169) Urban Women (n = 182) Rural women (n = 216).

**Fig 3** shows mean ASMI, grip strength and muscle function in men and women from urban and rural areas. ASMI and grip strength but not muscle function was higher in urban than rural men, whilst all three were higher in urban than rural women. For urban and rural men and rural women, greater age was associated with lower lean mass. Similar associations were observed for fat mass in rural men and women, and for bone mass in urban and rural women (all *p*<0.05).

### Prevalence of sarcopenia as per AWGS-2019 guidelines

We found that the overall prevalence of possible sarcopenia, sarcopenia and severe sarcopenia was 30.7%, 10.1%, and 4.2% respectively. Prevalence of sarcopenia and severe sarcopenia were higher in rural than urban participants (14.8% vs 5% and 6.8% vs 1.4% respectively, *p*<0.05 for both). Sarcopenia was also more common in men than women i.e.,12.5% vs 8% (*p*<0.05), although severe sarcopenia did not differ by sex (4.3% in men, 4% in women, (*p*>0.05).

In a subgroup analysis in which the study participants were divided into 4 subgroups as urban men, rural men and urban women, prevalence of sarcopenia was higher among rural men and women than their urban counterparts, i.e.,18.3% & 12.0% vs 6.8% & 3.3% (*p*<0.05). Similarly, prevalence of severe sarcopenia was higher among rural than urban men (6.5% vs 2.3%) and rural than urban women (6.9% vs 0.5%, both p<0.05). Possible sarcopenia was higher in rural than urban participants (35.1% vs 26.1%) and in men than women (27.2% vs 33.8%, both *p*<0.05). It was also higher among rural men than urban (36.1% vs 18.8%,) (*p*<0.05), but did not differ between rural and urban women (34.3% vs 33.2%) (*p*>0.05).

**Table 2** illustrates the model derived by binary logistic regression analysis which illustrates the association of sarcopenia and its determinants among study participants. No two or three-way-interactions between age, gender and location were observed (*p*>0.05 for all). Among factors studied, older age (OR = 1.098, (95% CI 1.064–1.133), p = 0.001), rural residence (OR = 2.456, (95% CI 1.291–4.670), p = 0.006), inadequate protein intake (OR = 2.042, (95% CI 1.063–3.921), p = 0.032), and lower socio-economic status (OR = 2.182, (95% CI 1.313–3.627, p = 0.003) were independently associated with sarcopenia risk. Tobacco consumption, sunlight exposure and physical activity were not significantly associated with sarcopenia in the whole group (*p*>0.05 for all).

**Table 2:** Association of sarcopenia and its determinants among study participants using binary logistic regression model.

Variables	Coefficient(B)	S.E.	Wald	P value	OR (95% CI)
Age	0.093	0.016	33.738	**0.001**	**1.098** (1.064–1.133)
Gender (Ref) Women	0.424	0.235	3.263	0.071	1.528 (0.965–2.420)
Location (Rural) (Ref) Urban	0.898	0.328	7.508	**0.006**	**2.456** (1.291–4.670)
Inadequate protein intake (Ref) Adequate protein intake	0.714	0.333	4.598	**0.032**	**2.042** (1.063–3.921)
Tobacco consumption (Ref) No tobacco	0.295	0.247	1.422	0.233	1.343(0.827–2.181)
Inadequate sunlight exposure (Ref) Adequate sunlight exposure	0.178	0.271	0.429	0.512	1.195 (0.702–2.034)
Lower socioeconomic class (Ref) Higher socio-economic class	0.780	0.259	9.072	**0.003**	**2.182 (**1.313–3.627)
Physically inactive (ref) Physically active	0.646	0.381	2.874	0.090	1.909(0.904–4.029)
Constant	-8.954	1.010	78.594	**0.000**	**0.000**

As prevalence of sarcopenia was higher in the rural participants we performed location-specific regression analysis to assess association of sarcopenia and related factors.

**Table 3** illustrates the regression analysis model derived separately for urban and rural participants. In the urban participants, older age (OR = 1.068 (95% CI 1.007–1.133), p = 0.028) and physical inactivity (OR = 4.587, (95%CI 1.529–13.760), p = 0.007) were found to be positively associated with sarcopenia. However, in the rural participants, older age (OR = 1.112, (95% CI 1.070–1.156), p = 0.001) and lower socio-economic status (OR = 2.150, (95% CI 1.214–3.808), p = 0.009) were significantly positively associated with sarcopenia.

**Table 3:** Location specific association between sarcopenia and its determinants by binary logistic regression model.

Variables	Coefficient(B)	S.E.	Wald	P value	OR (95% CI)
**Urban (n = 360)**					
Age	0.066	0.030	4.819	**0.028**	**1.068** (1.007–1.133)
Male gender (ref) female gender	0.655	0.496	1.747	0.186	1.925(0.729–5.086)
Inadequate protein intake (Ref) Adequate protein intake	0.792	0.508	2.431	0.119	2.209 (0.816–5.928)
Tobacco consumption (Ref) No tobacco consumption	0.041	0.666	0.004	0.951	1.042 (0.283–3.840)
Inadequate sunlight exposure (Ref) Adequate sunlight exposure	-0.774	0.843	0.844	0.358	0.461 (0.088–2.405)
Lower socioeconomic class (Ref) Higher socio-economic class	0.717	0.581	1.522	0.217	2.048 (0.656–6.395)
Physically inactive (Ref) Physically active	1.523	0.560	7.386	**0.007**	**4.587** (1.529–13.760)
Constant	-6.820	1.878	13.185	**0.000**	0.001
**Rural (n = 385)**					
Age	0.106	0.020	28.987	**0.001**	**1.112** (1.070–1.156)
Male gender (ref) female gender	0.365	0.277	1.740	0.187	1.441 (0.837–2.479)
Inadequate protein intake (Ref) Adequate protein intake	0.793	0.477	2.762	0.097	2.211 (0.867–5.636)
Tobacco consumption (Ref) No tobacco consumption	0.358	0.272	1.723	0.189	1.430 (0.838–2.439)
Inadequate sunlight exposure (Ref) Adequate sunlight exposure	0.323	0.285	1.290	0.256	1.382 (0.791–2.414)
Lower socioeconomic class (Ref) Higher socio-economic class	0.766	0.292	6.894	**0.009**	**2.150 (**1.214–3.808)
Physically inactive (Ref) Physically active	-0.032	0.507	0.000	0.994	1.004 (0.371–2.714)
Constant	-5.510	1.092	25.464	**0.000**	0.004

## Discussion

Our study investigated the prevalence of sarcopenia among middle-aged, community-dwelling adults from urban and rural areas in Pune, Western India. Using AWGS-2019 criteria and DXA-derived ASMI, the total prevalence of sarcopenia was 10% and severe sarcopenia was 4.2%. Prevalence of sarcopenia increased with age, was higher in men, and was more than twice as prevalent in rural areas than urban. In addition to age and rural residence, low protein intake and lower socioeconomic status were found to be associated with sarcopenia.

Only a small number of Indian studies have examined sarcopenia in community-based studies using more accurate DXA-based measurements. Limitations of previous studies include recruitment of hospital outpatients (Rahman et al from Vellore) [17], (Sreepriya et al from Thiruvananthapuram) [33] which may not be representative of the broader population, and anthropometry-based assessments of sarcopenia (Shaikh et.al) [34] from Karnataka. Another study by Tamilselvi et al from a district from Tamilnadu South India assessed the prevalence of sarcopenia using SARC-F questionnaire [18].

Using DXA derived-ASMI and AWGS criteria, we report the total prevalence of sarcopenia as 10.2% with 6.8% in urban and 14.8% in rural areas. The most comparable study examined sarcopenia in individuals of similar age (men 53.6±6.0y, women 50.1±4.6y) using DXA and AWGS criteria, finding 27% sarcopenia incidence [19]. This was the multicentre study conducted in different cohorts across different regions of India. In a study from urban Chandigarh-(North India) in individuals aged 20-85y DXA derived ASMI and EWGSOP prevalence of sarcopenia was 3.2% although the lower incidence is likely explained by lower mean participant age (44.4±15.4y) [35].

Ours is the first study to examine prevalence of sarcopenia across urban and rural populations in India. From the Asian subcontinent, we found only two studies which have compared prevalence of sarcopenia among urban and rural populations and used less accurate anthropometry (calf circumference) [36] from China and BIA measurements [37] from Korea. Our findings of a higher prevalence of sarcopenia in rural (21.6%) than urban (6.4%) areas are similar to these limited observations. In China, Gao et al.’s study reported total prevalence of sarcopenia as 9.8% with 13.1% in rural and 7% in urban areas, using AWGS criteria and calf circumference measurement [36]. Similarly in Korea, Moon et al. found sarcopenia prevalence rates of 24.4% and 16.3%, in rural and urban participants respectively using BIA-derived ASMI and AWGS criteria [16]. In agreement with the above-mentioned studies in other Asian countries, our study showed higher prevalence of low muscle mass, low grip strength, and low muscle function in rural compared to the urban participants.

We found a higher prevalence of sarcopenia in men than women, both in urban and rural areas (6.8% and 3.3% Vs 18.3% and 12% respectively). A recent global meta-analysis including data from around 700,000 individuals had contrasting findings of sex differences in sarcopenia dependent on the diagnostic criteria used. Studies using European Working Group on Sarcopenia in Older People 2 (EWGSOP2) found higher prevalence in men than women (11% vs 2%), whereas the opposite was observed in those employing International Working Group on Sarcopenia criteria (17% vs 12%). This may reflect the latter’s use of gait speed rather than hand grip as a primary diagnostic test, which would be influenced by higher fat percentage in women [37]. Based on systematic reviews from Asia and other countries, it has been observed that sarcopenia was more common among Asian men than women [36]. A study from Vellore (India) also reported higher prevalence in men than women i.e., 47% vs 23% in an older population attending a geriatric outpatient clinic [17]. However, in contrary to our results, a South Indian study in rural individuals aged 60 and over reported higher prevalence in women than men (24.5% vs 3.4%) although this was based only on anthropometric measurement [34]. A Chinese community-based study also reported higher prevalence of 19.2% in men vs 8.6% in women [38].

A methodological contributor to our finding of higher sarcopenia prevalence in men could be the use of hand grip strength as a primary diagnostic criterion as described above. Largely, alternatively, it could relate to higher physical activity levels in the presence of low protein intake in men observed in this study, or higher levels of tobacco consumption. Hormonal differences could also contribute, namely lower levels of IGF-1 which is an important mediator of muscle growth and repair Previous work found higher IGF-1 levels among women than men at a relatively younger age. As age advanced, the IGF-1 levels did not show an age-related decline in women, however, in men, IGF-1 showed an age-related decline [39]. Similar trends were observed in a small group of Caucasian and Chinese elderly suggesting that changes in IGF-1 may contribute to observed sex differences [17].

Higher prevalence of sarcopenia in rural residents indicates populations in rural areas are more vulnerable to sarcopenia than in urban areas. Variations in demographic profiles, body composition, nutritional status, lifestyle, and other cultural practices have an influence on peak skeletal muscle mass and rate of decline of muscle mass, strength, and function [17]. These and other environmental factors like access to food and healthcare facilities are likely to contribute to observed regional differences in sarcopenia risk.

In our study, lower socioeconomic class was also associated with increased risk of sarcopenia in rural participants. Low socio-economic status could be a cause of malnutrition in rural areas due to lack of accessible food resources and purchasing ability by rural individuals. Some Indian studies have found greater risk of sarcopenia in the lower socio-economic class, concurrent with low education and malnutrition Studies from Kolkata, India [11] and China [40] report that low education and income were associated with sarcopenia.

We also found that low protein intake was associated with increased risk of sarcopenia in rural participants. A Korean study found that lower intake of protein, energy and other micronutrients increases the risk of sarcopenia and inadequate protein intake may lead to loss of muscle mass and function [41]. Several other cohort studies have also found similar associations between protein and sarcopenia [42]. Another study by Kim et al revealed that, lower protein intake led to reduced muscle protein synthesis and subsequently affects net protein balance in older adults, which may explain these associations [43].

Numerous systematic reviews and meta-analyses report that physical inactivity is a risk factor for sarcopenia. A study by Mijnarends from the Netherlands found that in physically inactive individuals, incidence of sarcopenia was significantly higher as compared to more active individuals indicating that physical inactivity hastens the onset of sarcopenia [44]. As along with low protein intake, physical inactivity results in lower muscle protein synthesis rates [45]. Despite heavy physical activity and adequate sunlight exposure, we observed higher prevalence of sarcopenia among rural residents possibly indicating that diet and socio-economic status may overshadow the impact of physical activity in determining the risk of sarcopenia.

As India’s population is ageing rapidly, it is predicted that by 2041, it will be home to around 230 million elderly people [35]. With this increasing number, sarcopenia is likely to become a major public health concern in older adults leading to increased healthcare expenditure. There is thus an urgent need to plan strategies for early diagnosis and management of sarcopenia, which can be achieved by community screening of middle-aged and older individuals with simple and cost-effective screening tools and thus improving sarcopenia by appropriate interventions related to nutrition and physical activity [46].

Our study has several strengths; the study provides insights into the problem of sarcopenia from urban and rural areas of western India. This is the first Indian study to describe urban-rural differences in prevalence and factors associated with sarcopenia. We have used DXA which is now the gold standard to derive ASMI for diagnosis of low muscle mass. In most previous community-based studies from India, BIA, anthropometry, or other measures have been used due to their feasibility and low cost. Also, we have used SPPB which is a well-established and validated tool to assess muscle function.

One of the limitations of our study is that it is cross-sectional, hence we cannot comment on the causality. Longitudinal studies are needed for establishing causal relationships between sarcopenia and associated factors. As there is a lack of normative data in Indian population there are no India-specific cut-offs on muscle parameters, Further, we did not assess biochemical parameters like vitamin D, fasting insulin, IGF-1 etc due to logistics involved.

## Conclusion

This study assessed the prevalence and investigated the factors associated with sarcopenia among urban and rural Indian adults, and revealed much higher prevalence in rural individuals than their urban counterparts, and higher prevalence in men as compared to women. In this study, along with increasing age, lower socio-economic status and low protein intake were found to be significant risk factors for sarcopenia. Whilst these exposures partly explain rural-urban differences, additional influences such as lifestyle (physical activity, tobacco consumption) and other cultural factors may contribute to the remaining increased risk. Given the growing elderly population in India and increasing prevalence of sarcopenia, there is an urgent need to plan strategies for early identification, treatment, and management. Further integrated research and collaboration are required for standardization of techniques used for diagnosis of sarcopenia for achieving consistency in research in sarcopenia assessment.

## Supporting information

S1 Checklist (DOCX)
